# Antimicrobial Activity of a Novel Vascular Access Film Dressing Containing Chlorhexidine Gluconate

**DOI:** 10.1371/journal.pone.0143035

**Published:** 2015-11-23

**Authors:** Anne Wibaux, Priyaleela Thota, Jozef Mastej, Daniel L. Prince, Neal Carty, Peter Johnson

**Affiliations:** 1 Vancive Medical Technologies, an Avery Dennison business, 20 N. Wacker Drive Suite 2240, Chicago, Illinois, 60606, United States of America; 2 Gibraltar Laboratories Inc., Fairfield, New Jersey, 07004, United States of America; Institut Pasteur, FRANCE

## Abstract

**Background:**

Covering insertion sites with chlorhexidine impregnated dressings has been proven to be clinically effective in reducing catheter related blood stream infections (CR-BSI). Two chlorhexidine gluconate (CHG)-impregnated dressings are commercially available, a polyurethane foam disk and a film dressing containing a chlorhexidine gluconate-impregnated gel pad. While both have demonstrated efficacy in clinical settings, the major drawback of high cost and impaired IV insertion site visibility limits their usage. A new, simple film dressing containing CHG within its adhesive layer is now available. The objective of this study was to test the *in vitro* antimicrobial efficacy of the new dressing in comparison to the CHG-impregnated gel dressing.

**Methods:**

Quantitative aliquots of suspensions (concentration of 1.0x10^6^ to 5.0x10^6^ cfu/sample) of clinically relevant challenge organisms (*Staphylococcus* species, gram-negative bacilli, *Candida albicans*) were incubated in contact with the new CHG-containing film dressing, a placebo version of the same (negative control) and the commercially available CHG-impregnated gel dressing (positive control). Serial dilutions of the surviving organisms were quantified using the pour plate after 1, 3, 5, and 7 days of incubation in order to calculate an antimicrobial log_10_ reduction for each organism/dressing combination at each point in time.

**Results:**

The new CHG-containing film dressing delivered greater than 5.0 log_10_ reduction throughout the 7 days on all aerobic gram-negative bacilli and *Staphylococcus* species tested. As of day 1 the CHG-containing film dressing provided greater than 5.0 log_10_ reduction on *Candida albicans*. There were no statistically significant differences in the log_10_ reduction between the two dressings tested.

**Conclusion:**

The new CHG-containing film dressing was found to be as effective as the chlorhexidine gluconate-impregnated gel dressing on clinically relevant microbes.

## Background

Venous access via catheter insertion is a common practice in the hospital and outpatient settings. It is estimated that 30% to 80% of patients receive a catheter during their hospital stay, corresponding to 150 million intravascular devices used every year in the United States [1]. This procedure is not without risk. Microorganisms that colonize the implanted device or contaminate the fluid pathway at the time of insertion or during its use can result in bloodstream infections. Intravascular devices are one of the most important causes of health care-associated bloodstream infection. There are an estimated 250,000 to 500,000 central venous catheter bloodstream infections per year, accounting for 670 million to 2.68 billion dollars in additional healthcare costs and 100,000 deaths annually in the United States [2–5]. To reduce costs, the Centers for Medicare and Medicaid Services (CMS) is no longer paying for the care of hospital-associated catheter-related bloodstream infections (CR-BSI) [6].

A wide variety of micro-organisms have been shown to be associated with CR-BSIs. Nevertheless, the main microorganisms involved, representing the bulk of the microflora responsible for both central venous catheter and peripheral catheter bloodstream infections, are *Staphylococci* species including *Staphylococcus epidermidis* and *Staphylococcus aureus* that commensally inhabit the human skin, as well as aerobic gram-negative bacilli and *Candida* species [6–10]. Over the last decade some of those bacteria have become more antibiotic resistant [11, 12], representing an enormous challenge to clinicians. Transmissible from asymptomatic carriers, such organisms spread easily within healthcare institutions. Some of them have become resistant to nearly all antibiotics including carbapenems, considered as the antibiotics of last resort. In the last 3 years, an outbreak of Carbapenem-resistant Enterobacteriaceae (CRE) infections amongst immuno-compromised patients resulted in a 50% mortality rate [13]. About 4% of short-stay hospitals and 18% of the long-term acute care hospitals had at least one serious case of CRE in 2012 [14]. Another antibiotic resistant bacteria of concern is Methicillin-resistant *Staphylococcus aureus* (MRSA). MRSA is associated with substantial morbidity, mortality and unresolved treatment issues. MRSA has been implicated in more than 10% of the cases of peripheral venous catheter bloodstream infection and more than 20% of central venous catheter bloodstream infections [10, 15, 16].

Due to reduction in reimbursement of hospital acquired bloodstream infections as well as the appearance of antibiotic resistant bacteria (which has a significant impact on hospital stay) optimal management and prevention of these infections have become priorities for most health care facilities [6].

The efficacy of chlorhexidine gluconate-impregnated dressings in reducing the incidence of CR-BSI has been clinically proven [17–20]. To date, two chlorhexidine gluconate-impregnated dressings have been available as vascular access site dressings, a polyurethane foam disk (Biopatch^®^, Ethicon, New Jersey, USA) and a film dressing containing a chlorhexidine gluconate-impregnated gel pad (Tegaderm™ CHG, 3M Health Care, Minnesota, USA). While both have demonstrated clinical efficacy, the major drawbacks of high cost and reduced visibility of the IV insertion site limit their use. An alternative Chlorhexidine Gluconate dressing is now available as a simple semi-permeable polyurethane transparent film dressing. This dressing, BeneHold™ CHG Transparent Film Dressing (Vancive™ Medical Technologies, Chicago, IL, USA), while containing chlorhexidine gluconate, has similar properties to a film dressing: simple to use, breathable, transparent and with a cost comparable to a standard film dressing currently used on insertion sites.

This study was conducted to establish a comprehensive assessment of BeneHold™ CHG Transparent Film Dressing *in vitro* antimicrobial activity against the major microorganisms responsible for CR-BSIs as well as two antibiotic resistant bacteria of concern within healthcare institutions: MRSA and CRE. The test was conducted over seven days to align with the Centers for Disease Control guideline recommendation of IV site dressing changes within 7 days. A placebo was used as a negative control and a CHG impregnated gel pad, whose efficacy has been clinically proven, was used as a positive control.

## Methods

All tests were performed independently by Gibraltar Laboratories Inc., Fairfield, NJ, USA.

### Tested Products

Three products were included in the study: BeneHold™ CHG Transparent Film Dressing a transparent film dressing containing 4% (w/w) chlorhexidine gluconate within the adhesive, Tegaderm™ CHG a chlorhexidine gluconate-impregnated gel pad containing 2%(w/w) chlorhexidine gluconate incorporated into a transparent semi-breathable polyurethane dressing (3M Health Care, St Paul, MN, USA) used as a positive control and a placebo dressing identical to BeneHold™ CHG Transparent Film Dressing, but without chlorhexidine gluconate, was used as a negative control. Before testing the samples were aseptically cut into square 3cm x 3cm pieces.

### Test Organisms

Bacterial strains representing the most common microorganisms associated with CR-BSIs [9] together with three antibiotic resistant strains were included in the study (Table 1).

**Table 1:** Bacterial and fungal strains tested.

	Organism
	*S*.*aureus* (ATCC6538)^#^
*Staphyloccoci*	*MRSA* (ATCC33591)*
	*S*.*epidermidis* (ATCC12228)
	*E*.*coli* (ATCC8739)
	*K*.*pneumoniae* (ATCC4352)
**Gram-Negative Bacilli**	*CRE* (BAA-1705)*
	*CRE* (BAA-1706)*
	*P*.*aeruginosa* (ATCC9027)
	*E*.*aerogenes* (ATCC13043)
***Candida* species**	*C*.*albicans* (ATCC10231)

^#^methicillin-susceptible

*multi-resistant

### Preparation of Challenge Organisms

Bacterial cultures were grown with Trypticase Soy Agar (TSA) at 32.5°C ± 2.5°C for 18 to 24 hours and then harvested with 10% Tryptic Soy Broth (TSB) to achieve a final concentration of colony forming units (cfu) between 1.0 x 10^7^cfu/ml to 5.0 x 10^7^cfu/ml.

*Candida albicans* was grown in Sabouraud Dextrose Agar (SDA) at 32.5°C ± 2.5°C for 18 to 24 hours and then harvested with 10% TSB to achieve a final concentration between 1.0 x 10^7^cfu/ml to 5.0 x 10^7^cfu/ml. Incubation was performed at 22.5°C ± 2.5°C for 3 to 7 days.

Enumeration of all challenge organisms was performed by ten-fold serial dilution of the suspension in sterile 0.85% physiological saline. 2 x 1.0 ml aliquots from each dilution were plated using TSA for bacteria and SDA for yeast. Plates were incubated at 32.5°C ± 2.5°C for 3 to 5 days for bacteria and at 22.5°C ± 2.5°C for 3 to 5 days for yeast.

The number of cfu on each plate was counted and the mean cfu per ml was determined, representing the concentration of the challenge organism.

### Sample Inoculation

Samples were inoculated under a Class 100 biological safety Cabinet. The samples were aseptically placed in a sterile petri dish with their release liner facing up. Using a sterile forceps the release liner was peeled off.

One hundred fifty (150) μl of the prepared challenge organism solution containing 1.0x10^7^cfu/ml to 5.0x10^7^cfu/ml organisms were inoculated onto the test samples to achieve a concentration of 1.0x10^6^ to 5.0x10^6^cfu/sample. The inoculated test samples were covered with a 2cm x 2cm sterile cover film. The cover film was used to prevent drying of the inoculum, to ensure that the inoculum remained in intimate contact with the test sample surface under the cover film and to ensure that the inoculum did not spread beyond the edges of the test samples. Each product, bacterium/yeast and time point combination was tested in triplicate.

The inoculated test samples were held at 37°C ± 1°C / 75% ± 5% relative humidity (RH) for the following contact times: 1 day, 3 days, 5 days and 7 days.

### Enumeration of Challenge Organisms after Incubation

After each contact time interval for each inoculated sample the cover film was carefully separated from the sample and placed into the same vessel containing 100ml of Dey/Engley (D/E) neutralizing broth. The vessels containing test samples were sonicated for 10 minutes. Enumeration of the recovered microorganisms was performed by the pour plate method using ten-fold serial dilutions. Incubation was done at 32.5°C ± 2.5°C for 48 to 72 hours for bacteria and 22.5°C ± 2.5°C for 5 to 7 days for yeast. The number of cfu on each plate was counted and the mean cfu per sample was determined, representing the recovery of the challenge organism.

### Log_10_ Reduction Calculation

The triplicate replicates of the test samples were averaged and transformed to log_10_cfu/sample. The log_10_ reduction to evaluate the effectiveness of the tested materials was calculated as the difference between the log_10_ of the inoculum and the log_10_ of the average cfu recovered from the tested samples.

### Chlorhexidine Gluconate Neutralization

To assure validity of the results, neutralization of the D/E broth was validated for BeneHold™ CHG Transparent Film Dressing, Tegaderm™ CHG and the placebo for a representative microorganism of each species. Bacterial cultures were grown with Trypticase Soy Agar (TSA) at 32.5°C ± 2.5°C for 18 to 24 hours and then harvested and diluted with 0.85% physiological saline to obtain a final concentration of 1000 cfu/ml. *Candida albicans* was grown in Sabouraud Dextrose Agar (SDA) at 32.5°C ± 2.5°C for 18 to 24 hours and then harvested and diluted with 0.85% physiological saline to obtain a final concentration of 1000 cfu/ml.

The test article count was obtained by inoculating 150 μl of the prepared challenge microorganisms onto the test articles (BeneHold™ CHG Transparent Film Dressing, Tegaderm™ CHG and the placebo) in triplicate. Within 10 minutes the inoculated samples were placed in vessel containing 100ml of D/E broth and sonicated for 10 minutes. The inoculum count was confirmed by placing 150 μl of the prepared challenge organism in 10ml of 0.85% physiological saline. The toxicity control was obtained by inoculating 150 μl of the prepared challenge microorganisms directly into D/E broth. Test article count, inoculum count and toxicity control enumeration of the recovered microorganisms were performed by the pour plate method using ten-fold serial dilutions.

The following were calculated: Neutralizer Efficacy (D) = average test sample / average toxicity control, Neutralizer toxicity (E) = average toxicity control / average inoculum count, microbial recovery (F) = average test sample / average inoculum count. The neutralization recovery was considered validated when the results of all 3 groups were greater than 0.7.

### Statistical Analysis

Data was analysed using the nonparametric Mann-Whitney test. A P value ≤0.05 was considered to be significant.

## Results

The bacterial count of greater than 6.0 log_10_ cfu on the placebo from day 1 to day 7 (Table 2) demonstrated that the experimental conditions, preparation of the samples, inoculum composition and incubation were adequate in that they allowed the test organisms to survive. Therefore, the microbial reduction observed on the BeneHold™ CHG Transparent Film Dressingis attributed to the presence of chlorhexidine gluconate and not to the nature of the base adhesive or the experimental conditions.

**Table 2:** *In vitro* microbial challenge over 7 days.

Test organisms		BeneHold™ CHG Transparent Film Dressing					Tegaderm™ CHG					Placebo				
		Day0	Day1	Day3	Day5	Day7	Day0	Day1	Day3	Day5	Day7	Day0	Day1	Day3	Day5	Day7
		*4*.*8x10* ^*6*^	*<10*	*<10*	*<10*	*<10*	*2*.*3x10* ^*6*^	*<10*	*<10*	*<10*	*<10*	*4*.*8x10* ^*6*^	*4*.*4x10* ^*7*^	*4*.*0x10* ^*6*^	*2*.*4x10* ^*6*^	*7*.*1x10* ^*6*^
***MRSA***	*CFU*	*3*.*7x10* ^*6*^	*<10*	*<10*	*<10*	*<10*	*2*.*0x10* ^*6*^	*10*	*<10*	*<10*	*<10*	*3*.*7x10* ^*6*^	*4*.*1x10* ^*7*^	*4*.*9x10* ^*6*^	*6*.*5x10* ^*6*^	*5*.*1x10* ^*6*^
***ATCC33591***		*4*.*8x10* ^*6*^	*<10*	*<10*	*<10*	*<10*	*2*.*0x10* ^*6*^	*35*	*<10*	*<10*	*<10*	*4*.*8x10* ^*6*^	*5*.*1x10* ^*7*^	*8*.*0x10* ^*6*^	*6*.*1x10* ^*6*^	*3*.*1x10* ^*6*^
	***Average***	***4*.*4x10*** ^***6***^	***<10***	***<10***	***<10***	***<10***	***2*.*1x10*** ^***6***^	***18***	***<10***	***<10***	***<10***	***4*.*4x10*** ^***6***^	***4*.*5x10*** ^***7***^	***5*.*6x10*** ^***6***^	***5*.*0x10*** ^***6***^	***5*.*1x10*** ^***6***^
		*4*.*7x10* ^*6*^	*<10*	*<10*	*<10*	*<10*	*3*.*6x10* ^*6*^	*<10*	*<10*	*<10*	*<10*	*4*.*7x10* ^*6*^	*8*.*8x10* ^*6*^	*1*.*2x10* ^*6*^	*1*.*4x10* ^*6*^	*5*.*0x10* ^*6*^
***S*.*aureus***	*CFU*	*4*.*2x10* ^*6*^	*<10*	*<10*	*<10*	*<10*	*3*.*6x10* ^*6*^	*<10*	*<10*	*<10*	*<10*	*4*.*2x10* ^*6*^	*9*.*7x10* ^*6*^	*1*.*0x10* ^*6*^	*2*.*2x10* ^*6*^	*5*.*0x10* ^*6*^
***ATCC6538***		*5*.*0x10* ^*6*^	*<10*	*<10*	*<10*	*<10*	*4*.*0x10* ^*6*^	*<10*	*<10*	*<10*	*<10*	*5*.*0x10* ^*6*^	*8*.*2x10* ^*6*^	*1*.*2x10* ^*6*^	*1*.*5x10* ^*6*^	*8*.*1x10* ^*6*^
	***Average***	***4*.*6x10*** ^***6***^	***<10***	***<10***	***<10***	***<10***	***3*.*7x10*** ^***6***^	**<10**	**<10**	**<10**	**<10**	***4*.*6x10*** ^***6***^	***8*.*9x10*** ^***6***^	***1*.*1x10*** ^***6***^	***1*.*7x10*** ^***6***^	***6*.*0x10*** ^***6***^
		*3*.*6x10* ^*6*^	*<10*	*<10*	*<10*	*<10*	*5*.*0x10* ^*6*^	*<10*	*<10*	*<10*	*<10*	*3*.*6x10* ^*6*^	*5*.*7x10* ^*7*^	*1*.*3x10* ^*6*^	*3*.*7x10* ^*5*^	*3*.*2x10* ^*6*^
***S*.*epidermidis***	*CFU*	*4*.*9x10* ^*6*^	*<10*	*<10*	*<10*	*<10*	*5*.*0x10* ^*6*^	*<10*	*<10*	*<10*	*<10*	*4*.*9x10* ^*6*^	*4*.*6x10* ^*7*^	*1*.*4x10* ^*6*^	*2*.*0x10* ^*6*^	*8*.*5x10* ^*5*^
***ATCC12228***		*5*.*0x10* ^*6*^	*<10*	*<10*	*<10*	*<10*	*4*.*7x10* ^*6*^	*<10*	*<10*	*<10*	*<10*	*5*.*0x10* ^*6*^	*7*.*7x10* ^*7*^	*1*.*5x10* ^*6*^	*3*.*6x10* ^*6*^	*8*.*8x10* ^*5*^
	***Average***	***4*.*5x10*** ^***6***^	***<10***	***<10***	***<10***	***<10***	***4*.*9x10*** ^***6***^	***<10***	***<10***	***<10***	***<10***	***4*.*5x10*** ^***6***^	***6*.*0x10*** ^***7***^	***1*.*4x10*** ^***6***^	***2*.*0x10*** ^***6***^	***1*.*6x10*** ^***6***^
		*4*.*9x10* ^*6*^	*<10*	*<10*	*<10*	*<10*	*4*.*4x10* ^*6*^	*<10*	*<10*	*<10*	*<10*	*4*.*9x10* ^*6*^	*1*.*3x10* ^*8*^	*9*.*0x10* ^*7*^	*1*.*5x10* ^*8*^	*8*.*4x10* ^*7*^
***E*.*coli***	*CFU*	*4*.*4x10* ^*6*^	*<10*	*<10*	*<10*	*<10*	*4*.*0x10* ^*6*^	*<10*	*<10*	*<10*	*<10*	*4*.*4x10* ^*6*^	*1*.*6x10* ^*8*^	*8*.*7x10* ^*7*^	*1*.*1x10* ^*8*^	*9*.*4x10* ^*7*^
***ATCC8739***		*4*.*6x10* ^*6*^	*<10*	*<10*	*<10*	*<10*	*4*.*5x10* ^*6*^	*<10*	*<10*	*<10*	*<10*	*4*.*6x10* ^*6*^	*1*.*2x10* ^*8*^	*9*.*7x10* ^*7*^	*1*.*2x10* ^*8*^	*7*.*4x10* ^*7*^
	***Average***	***4*.*6x10*** ^***6***^	***<10***	***<10***	***<10***	***<10***	***4*.*3x10*** ^***6***^	***<10***	***<10***	***<10***	***<10***	***4*.*6x10*** ^***6***^	***1*.*4x10*** ^***8***^	***9*.*1x10*** ^***7***^	***1*.*3x10*** ^***8***^	***8*.*4x10*** ^***7***^
		*3*.*5x10* ^*6*^	*<10*	*<10*	*<10*	*<10*	*4*.*6x10* ^*6*^	*<10*	*<10*	*<10*	*<10*	*3*.*5x10* ^*6*^	*1*.*0x10* ^*8*^	*1*.*2x10* ^*8*^	*3*.*9x10* ^*8*^	*3*.*3x10* ^*8*^
***P*.*aeruginosa***	*CFU*	*4*.*0x10* ^*6*^	*<10*	*<10*	*<10*	*<10*	*4*.*7x10* ^*6*^	*<10*	*<10*	*<10*	*<10*	*4*.*0x10* ^*6*^	*1*.*2x10* ^*8*^	*1*.*3x10* ^*8*^	*2*.*2x10* ^*8*^	*4*.*1x10* ^*8*^
***ATCC9027***		*3*.*7x10* ^*6*^	*<10*	*<10*	*<10*	*<10*	*4*.*0x10* ^*6*^	*<10*	*<10*	*<10*	*<10*	*3*.*7x10* ^*6*^	*1*.*3x10* ^*8*^	*1*.*3x10* ^*8*^	*4*.*4x10* ^*8*^	*3*.*5x10* ^*8*^
	***Average***	***3*.*7x10*** ^***6***^	***<10***	***<10***	***<10***	***<10***	***4*.*4x10*** ^***6***^	***<10***	***<10***	***<10***	***<10***	***3*.*7x10*** ^***6***^	***1*.*2x10*** ^***8***^	***1*.*3x10*** ^***8***^	***3*.*5x10*** ^***8***^	***3*.*6x10*** ^***8***^
		*4*.*4x10* ^*6*^	*<10*	*<10*	*<10*	*<10*	*1*.*5x10* ^*6*^	*<10*	*<10*	*<10*	*<10*	*4*.*4x10* ^*6*^	*1*.*6x10* ^*8*^	*7*.*4x10* ^*7*^	*4*.*7x10* ^*7*^	*1*.*0x10* ^*6*^
***K*.*pneumoniae***	*CFU*	*5*.*0x10* ^*6*^	*<10*	*<10*	*<10*	*<10*	*2*.*7x10* ^*6*^	*<10*	*<10*	*<10*	*<10*	*5*.*0x10* ^*6*^	*1*.*5x10* ^*8*^	*8*.*7x10* ^*7*^	*6*.*1x10* ^*7*^	*1*.*1x10* ^*6*^
***ATCC4352***		*4*.*9x10* ^*6*^	*<10*	*<10*	*<10*	*<10*	*1*.*6x10* ^*6*^	*<10*	*<10*	*<10*	*<10*	*4*.*9x10* ^*6*^	*1*.*4x10* ^*8*^	*1*.*4x10* ^*8*^	*7*.*7x10* ^*7*^	*1*.*0x10* ^*6*^
	***Average***	***4*.*8x10*** ^***6***^	***<10***	***<10***	***<10***	***<10***	***1*.*9x10*** ^***6***^	***<10***	***<10***	***<10***	***<10***	***4*.*8x10*** ^***6***^	***1*.*5x10*** ^***8***^	***1*.*0x10*** ^***8***^	***6*.*2x10*** ^***7***^	***1*.*0x10*** ^***6***^
		*4*.*2x10* ^*6*^	***25***	*<10*	*<10*	*<10*	*4*.*2x10* ^*6*^	*<10*	*<10*	*<10*	*<10*	*4*.*2x10* ^*6*^	*1*.*2x10* ^*8*^	*1*.*1x10* ^*8*^	*7*.*5x10* ^*7*^	*3*.*5x10* ^*7*^
***CRE BAA-1705***	*CFU*	*3*.*0x10* ^*6*^	***35***	*<10*	*<10*	*<10*	*3*.*0x10* ^*6*^	*<10*	*<10*	*<10*	*<10*	*3*.*0x10* ^*6*^	*8*.*1x10* ^*7*^	*1*.*0x10* ^*8*^	*7*.*5x10* ^*7*^	*4*.*7x10* ^*7*^
		*3*.*8x10* ^*6*^	***25***	***<10***	***<10***	***<10***	*3*.*8x10* ^*6*^	***<10***	***<10***	***<10***	***<10***	*3*.*8x10* ^*6*^	*1*.*3x10* ^*8*^	*1*.*1x10* ^*8*^	*3*.*2x10* ^*7*^	*4*.*1x10* ^*7*^
	***Average***	***3*.*7x10*** ^***6***^	***28***	***<10***	***<10***	***<10***	***3*.*7x10*** ^***6***^	***<10***	***<10***	***<10***	***<10***	***3*.*7x10*** ^***6***^	***1*.*1x10*** ^***8***^	***1*.*1x10*** ^***8***^	***6*.*1x10*** ^***7***^	***4*.*1x10*** ^***7***^
		*1*.*0x10* ^*6*^	*<10*	*<10*	*<10*	*<10*	*1*.*0x10* ^*6*^	*<10*	*<10*	*<10*	*<10*	*1*.*0x10* ^*6*^	*7*.*4x10* ^*7*^	*7*.*1x10* ^*7*^	*3*.*4x10* ^*7*^	*2*.*2x10* ^*7*^
***CRE BAA-1706***	*CFU*	*1*.*2x10* ^*6*^	*<10*	*<10*	*<10*	*<10*	*1*.*2x10* ^*6*^	*<10*	*<10*	*<10*	*<10*	*1*.*2x10* ^*6*^	*1*.*4x10* ^*8*^	*7*.*6x10* ^*7*^	*3*.*6x10* ^*7*^	*9*.*1x10* ^*6*^
		*1*.*1x10* ^*6*^	***<10***	***<10***	***<10***	***<10***	*1*.*1x10* ^*6*^	***<10***	***<10***	***<10***	***<10***	*1*.*1x10* ^*6*^	*1*.*1x10* ^*8*^	*8*.*0x10* ^*7*^	*3*.*1x10* ^*7*^	*1*.*4x10* ^*7*^
	***Average***	***1*.*1x10*** ^***6***^	***<10***	***<10***	***<10***	***<10***	***1*.*1x10*** ^***6***^	***<10***	***<10***	***<10***	***<10***	***1*.*1x10*** ^***6***^	***1*.*1x10*** ^***8***^	***7*.*6x10*** ^***7***^	***3*.*4x10*** ^***7***^	***1*.*4x10*** ^***7***^
		*4*.*9x10* ^*6*^	*<10*	*<10*	*<10*	*<10*	*4*.*9x10* ^*6*^	*<10*	*<10*	*<10*	*<10*	*5*.*0x10* ^*6*^	*6*.*7x10* ^*8*^	*3*.*8x10* ^*8*^	*9*.*5x10* ^*7*^	*1*.*7x10* ^*8*^
***E*.*aerogenes***	*CFU*	*4*.*3x10* ^*6*^	*<10*	*<10*	*<10*	*<10*	*4*.*9x10* ^*6*^	*<10*	*<10*	*<10*	*<10*	*4*.*7x10* ^*6*^	*6*.*7x10* ^*8*^	*3*.*5x10* ^*8*^	*1*.*2x10* ^*8*^	*2*.*4x10* ^*8*^
***ATCC13043***		*5*.*0x10* ^*6*^	***<10***	***<10***	***<10***	***<10***	*4*.*7x10* ^*6*^	***<10***	***<10***	***<10***	***<10***	*4*.*7x10* ^*6*^	*6*.*8x10* ^*8*^	*4*.*1x10* ^*8*^	*9*.*0x10* ^*7*^	*1*.*7x10* ^*8*^
	***Average***	***4*.*7x10*** ^***6***^	***<10***	***<10***	***<10***	***<10***	***4*.*8x10*** ^***6***^	***<10***	***<10***	***<10***	***<10***	***4*.*8x10*** ^***6***^	***6*.*7x10*** ^***8***^	***3*.*8x10*** ^***8***^	***1*.*0x10*** ^***8***^	***1*.*9x10*** ^***8***^
		*1*.*0x10* ^*6*^	*<10*	*<10*	*<10*	*<10*	*3*.*2x10* ^*6*^	*1*.*5x10* ^*2*^	*<10*	*<10*	*<10*	*1*.*0x10* ^*6*^	*1*.*3x10* ^*6*^	*7*.*7x10* ^*4*^	*1*.*2x10* ^*5*^	*1*.*9x10* ^*5*^
***C*.*albicans***	*CFU*	*1*.*2x10* ^*6*^	*<10*	*<10*	*<10*	*<10*	*5*.*9x10* ^*6*^	*3*.*4x10* ^*2*^	*<10*	*<10*	*<10*	*1*.*2x10* ^*6*^	*5*.*6x10* ^*5*^	*2*.*2x10* ^*5*^	*2*.*0x10* ^*5*^	*1*.*7x10* ^*5*^
***ATCC10231***		*1*.*2x10* ^*6*^	*<10*	*<10*	*<10*	*<10*	*5*.*1x10* ^*6*^	*3*.*1x10* ^*2*^	*<10*	*<10*	*<10*	*1*.*2x10* ^*6*^	*8*.*3x10* ^*5*^	*1*.*9x10* ^*5*^	*8*.*1x10* ^*4*^	*1*.*9x10* ^*5*^
	***Average***	***1*.*1x10*** ^***6***^	***<10***	***<10***	***<10***	***<10***	***4*.*7x10*** ^***6***^	***2*.*7x10*** ^***2***^	***<10***	***<10***	***<10***	***1*.*1x10*** ^***6***^	***9*.*0x10*** ^***5***^	***1*.*6x10*** ^***5***^	***1*.*3x10*** ^***5***^	***1*.*8x10*** ^***5***^

CFU = colony forming units; MRSA = methicillin-resistant *staphylococcus aureus*; CRE = Carbapenem-resistant *enterobacteriaceae*

From day 1 throughout day 7, both BeneHold™ CHG Transparent Film Dressing and Tegaderm™ CHG showed greater than 5.0 log_10_ reduction on all aerobic gram-negative bacilli tested (Table 3). There was no statistically significant difference between the two products (*p*> 0.86).

**Table 3:** Mean log_10_ reductions measured for each microorganism at each point in time.

	BeneHold™ CHG Transparent Film Dressing				Tegaderm™ CHG			
Test organisms	Day1	Day3	Day5	Day7	Day1	Day3	Day5	Day7
*MRSA (ATCC33591)*	>5.0	>5.0	>5.0	>5.0	>5.0	>5.0	>5.0	>5.0
*S*.*aureus(ATCC6538)*	>5.0	>5.0	>5.0	>5.0	>5.0	>5.0	>5.0	>5.0
*S*.*epidermidis(ATCC12228)*	>5.0	>5.0	>5.0	>5.0	>5.0	>5.0	>5.0	>5.0
*E*.*coli (ATCC8739)*	>5.0	>5.0	>5.0	>5.0	>5.0	>5.0	>5.0	>5.0
*P*.*aeruginosa(ATCC9027)*	>5.0	>5.0	>5.0	>5.0	>5.0	>5.0	>5.0	>5.0
*K*.*pneumoniae(ATCC4352)*	>5.0	>5.0	>5.0	>5.0	>5.0	>5.0	>5.0	>5.0
*CRE (BAA1705)*	>5.0	>5.0	>5.0	>5.0	>5.0	>5.0	>5.0	>5.0
*CRE (BAA1706)*	>5.0	>5.0	>5.0	>5.0	>5.0	>5.0	>5.0	>5.0
*E*.*aerogenes(ATCC13043)*	>5.0	>5.0	>5.0	>5.0	>5.0	>5.0	>5.0	>5.0
*C*.*albicans(ATCC10231)*	>5.0	>5.0	>5.0	>5.0	3.24	>5.0	>5.0	>5.0

MRSA = methicillin-resistant *staphylococcus aureus*; CRE = Carbapenem-resistant *Enterobacteriaceae*

Against *Staphyloccocus aureus* species and *Staphyloccocus epidermidis*, BeneHold™ CHG Transparent Film Dressinggenerated more than 5.0 log_10_ reduction (Table 3). There was still 1.25 log_10_ cfu of MRSA remaining after 1 day of exposure to Tegaderm™ CHG (Table 2), however the difference between the two products was not statistically significant (*p* = 0.25).

BeneHold™ CHG Transparent Film Dressing was fully effective against *Candida albicans* as of day 1 with a greater than 5.0 log_10_ reduction, compared to a 3.2 log_10_ reduction for Tegaderm™ CHG (Table 3). The difference between the two products was not statistically significant (*p* = 0.08).

*S*. *aureus*, *E*. *coli* and *C*. *albicans* were selected as challenge microorganisms to conduct the D/E broth neutralization recovery validation. Neutralizer efficacy, neutralizer toxicity, and microbial recovery were all found to meet the acceptance criteria; all were greater than 0.7 (Table 4). The D/E broth neutralization recovery was validated.

**Table 4:** Results from Chlorhexidine Gluconate neutralization assay.

	BeneHold™ CHG Transparent Film Dressing			Tegaderm™ CHG			Placebo		
	D	E	F	D	E	F	D	E	F
*S*.*aureus*	1.2	1.1	1.3	1.2	1.1	1.3	1.1	1.1	1.2
*E*.*coli*	1.0	1.0	1.0	1.0	1.0	1.1	1.0	1.0	1.0
*C*.*albicans*	1.1	1.0	1.1	1.1	1.0	1.1	1.0	1.0	1.0

D = % Neutralizer Efficacy, E = % Neutralizer toxicity, F = % Microbial recovery

## Discussion

This study demonstrated that the BeneHold™ CHG Transparent Film Dressing, a simple film dressing containing chlorhexidine gluconate within its adhesive has similar *in vitro* antimicrobial efficacy in the time-kill experiment as Tegaderm™ CHG against a representative set of microorganisms known for their high association with CR-BSIs. Both dressings showed rapid antimicrobial activity. The antimicrobial efficacy was maintained over 7 days.

In 2011, the Centers for Disease Control and Infection (CDC) evaluated clinical data from patients treated with CHG-containing Biopatch Protective Disk as part of its update to the 2002 guidelines for reducing risk of intravascular catheter-related infections. In the updated guidelines, use of a CHG-containing dressing is designated as a category 1B recommendation [21]. In a recent meta-analysis of 9 randomized controlled trials, chlorhexidine-impregnated dressings were shown to be beneficial in preventing catheter colonization and CR-BSI [22]. Use of a chlorhexidine-impregnated dressings resulted in a decreased prevalence of CR-BSIs (relative risk, 0.60; 95% CI, 0.41–0.88, p = 0.009). The prevalence of catheter colonization was also significantly reduced in the chlorhexidine-impregnated dressing group (relative risk, 0.52; 95% CI, 0.43–0.64; p < 0.001).

It is evident that adequate concentrations of antiseptics are required at a catheter insertion sites to prevent catheter colonization and reduce the risk of CR-BSIs. Emergence of antibiotic resistant microbial strains is a key concern for healthcare institutions as it complicates therapeutic management and increases costs of care. Not only the concentration, but also the availability, of antimicrobial agent within a dressing determines the antimicrobial activity. To avoid bacterial resistance, a dressing should release enough of an antimicrobial agent as to generate antimicrobial concentrations at least 10 times greater than the minimum inhibitory concentration (MIC) [23]. Within the context of this study, BeneHold™ CHG Transparent Film Dressing has been estimated to provide a maximum chlorhexidine gluconate concentration of 24,000μg/ml (Table 5) while Tegaderm provides a maximum concentration of 17,000μg/ml. These maximum chlorhexidine gluconate concentrations are more than one hundred to one thousand times greater than the MIC reported in the literature [24, 25]. This explains the full kill observed in this study for both dressings.

**Table 5:** Mean log_10_ reductions.

	BeneHold™ CHG Transparent Film Dressing	Tegaderm™ CHG
CHG content in a 3cm x 3cm sample	3.6mg	40.1mg
Volume of solution	0.150ml (inoculum)	0.150ml (inoculum) and 2.25cm^3^ of gel approximated to 2.15ml
CHG concentration in the experiment	3.6mg/150μl or24000μg/ml	40.1mg/2.3ml or 17335μg/ml

A prior *in vivo* study, assessing inhibition of native microflora under the Benehold™ CHG containing film dressing compared to a simple non antimicrobial dressing after day 1, 4 and 7 demonstrated a statistically significant difference between the two treatments after both day 4 and day 7. A significant decrease in bacterial count was shown with CHG containing film dressing while significant bacterial regrowth was shown with plain film dressing. Comparing the two, a difference greater than 3log_10_ cfu/cm^2^ was observed after 7 days (p = 0.001) [26].

Surprisingly, a prior *in vitro* time-kill study performed on the chlorhexidine gluconate-impregnated gel reported more than 6.0 log_10_ reduction for MRSA and *Escherichia coli* after only 5 minutes following inoculation [27]. In our study, MRSA was recovered from the chlorhexidine-impregnated gel after one day. The disparity in these results can be explained by more challenging conditions used in the present study. Karpanen *et al* [27] used 20 μl of a phosphate buffer saline as inoculum while 150 μl of a more challenging enriched inoculum containing 10% TSB was used in our protocol.

The favorable comparison between BeneHold™ CHG Transparent Film Dressing and Tegaderm™ CHG suggests the possibility that the thin film dressings may provide significant clinical benefit by combining antisepsis with the useful properties of film dressings. Ability to clearly visualize the entire vascular access site would enable care-givers to make an objective evaluation of whether a dressing change is needed. Thinness and conformability would potentially increase patient comfort and also make the dressings less prone to accidental removal due to edge lift.

### Limitations

This study was conducted in a non-living, non-disturbed environment. Conditions in clinical settings might be different. The efficacy of chlorhexidine gluconate in the dressing may be theoretically reduced by skin lipids and proteins. In addition, bacterial biofilm production could theoretically reduce efficacy. While this study has the potential limitation of being sponsored by industry (Vancive™ Medical Technologies), all tests were performed independently by Gibraltar Laboratories Inc.

## Conclusions

The new chlorhexidine gluconate-containing film dressing is highly effective against a variety of clinically relevant microorganisms. BeneHold™ CHG Transparent Film Dressing, showed similar *in vitro* antimicrobial efficacy in the time-kill experiments as Tegaderm™ CHG. Further studies are needed to establish the efficacy of CHG-containing thin film dressings against catheter-related blood stream infections in clinical settings.
